# Behçet's disease‐like clinical manifestations of chronic lymphocytic leukemia during good response to ibrutinib

**DOI:** 10.1002/jha2.752

**Published:** 2023-06-29

**Authors:** Kosuke Obama, Hana Yamamoto, Hirosaka Inoue

**Affiliations:** ^1^ Department of Hematology Imakiire General Hospital Kagoshima Japan

**Keywords:** Behçet's disease, CD11c, chronic lymphocytic leukemia, ibrutinib

1

A 68‐year‐old man was admitted to our hospital with chronic inflammatory polyneuropathy. On admission, he had an elevated white blood cell count of 32.6 × 10^9^/l, with 73% abnormal lymphocytes (Figure 1A). The lymphocytes were small cells with mild atypia, and flow cytometry analysis was positive for CD5, 19, 20, 21, 22, 25, and 11c, and negative for CD23. Based on these results, the patient was diagnosed with chronic lymphocytic leukemia (CLL).

**FIGURE 1:**
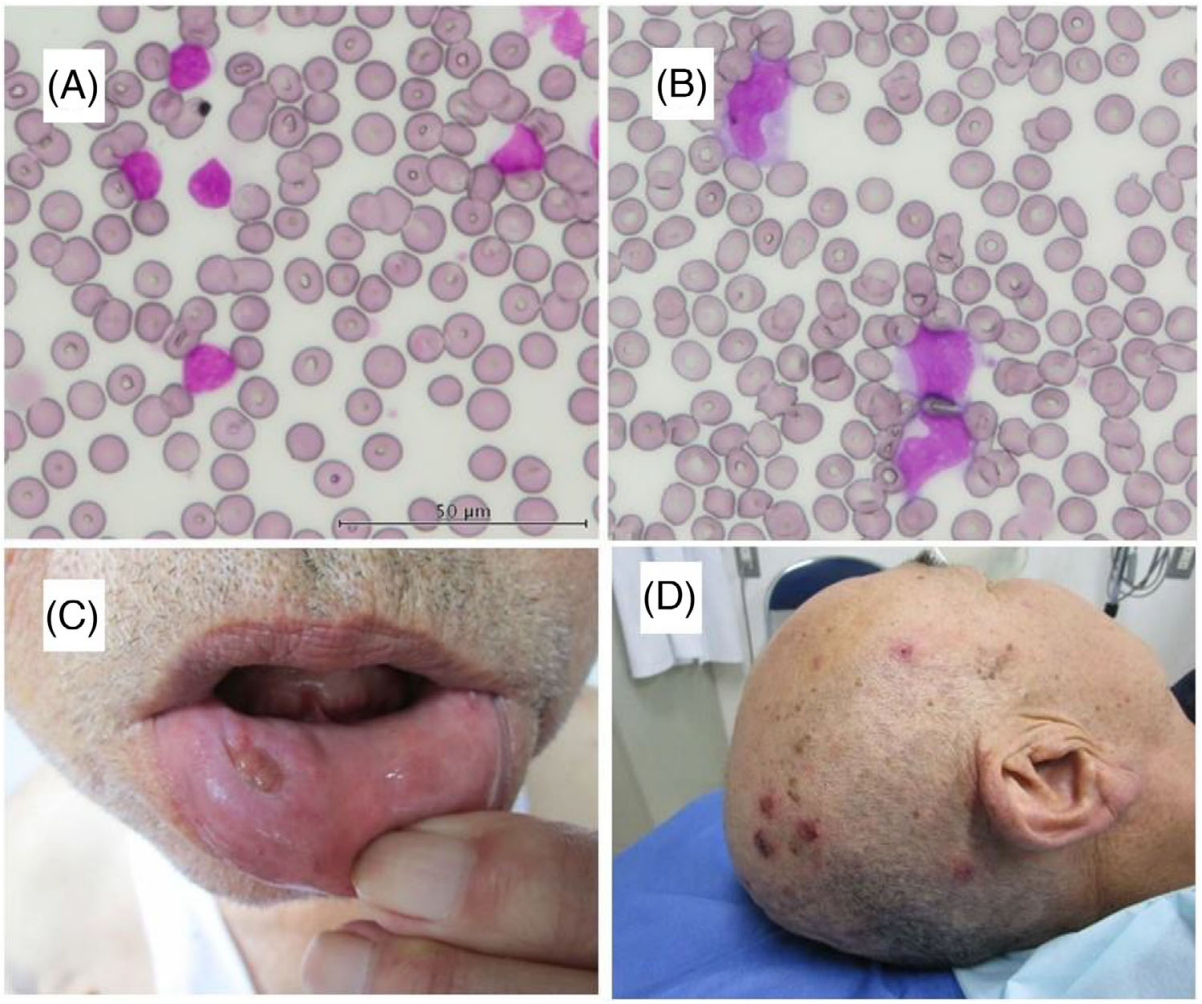
(A) Peripheral blood at the initial diagnosis; (B) peripheral blood at exacerbation of Behcet's disease‐like symptoms; (C and D) mucositis and folliculitis observed at exacerbation of Behcet's disease‐like symptoms.

Four years after diagnosis, the patient was admitted to our hospital because of abdominal pain. Computed tomography (CT) revealed lymph node enlargement in the abdominal cavity, without hepatosplenomegaly. The white blood cell count increased to 58.4 × 10^9^/l, with 68% abnormal lymphocytes. Ibrutinib was administered to the patient and a progressively good response was observed. Ten months after the ibrutinib administration, the patient was hospitalized for severe stomatitis, folliculitis, and vulvar ulceration (Figure 1C,D). The peripheral blood showed a small number of small lymphocytes with mild nuclear atypia, and laboratory findings showed no significant changes. His initial diagnosis was Behçet's disease (BD); however, skin and mucous membrane biopsy revealed abnormal B‐cell infiltration, which proved to be an infiltration of CLL. Two weeks after admission, large abnormal lymphocytes were observed in the peripheral blood (Figure 1B). Although the lymphocytic morphology was distinct from that at the initial diagnosis, the phenotype detected by flow cytometry was consistent with that at the initial diagnosis. CT revealed free air in the abdominal cavity caused by an asymptomatic gastrointestinal tract perforation. Ibrutinib administration was then switched to venetoclax, and subsequently, all the symptoms and laboratory findings, including abnormal lymphocytes in the peripheral blood, rapidly improved. After induction of venetoclax, CLL has been well controlled for more than 2 years.

In this case, mantle cell lymphoma (MCL) could not be ruled out because of the negative findings for CD23. Although detailed analysis has not been performed, MCL is considered negative based on the long‐term favorable clinical course and CD11c expression. And based on the major morphological changes in CLL cells during exacerbation of BD‐like clinical manifestations, we initially assumed CLL transformation. However, the characteristic localized clinical features and favorable course of the disease after venetoclax administration suggest that this process may have been a transient activation induced by ibrutinib. Ibrutinib has been reported to have various effects on the immune system, including a shift of T cells to Th1 dominance via the suppression of IL2‐inducible T‐cell kinase. BD is known to have a Th1 dominant immune background, and ibrutinib may influence CLL cell kinetics through these immunological activities. Furthermore, expression of CD11c, which has been the focus of attention in BD disease, may be associated with these specific clinical manifestations.

Ibrutinib has been reported to induce various adverse effects, many of which resemble BD symptoms. Recently, some cases of CLL with uveitis treated with ibrutinib have been reported. The effects of ibrutinib on the immune system may be closely related to the immunopathology of BD.

## AUTHOR CONTRIBUTIONS

Planning and conducting research and writing manyuscript: Kosuke Obama. Treatment co‐responsibility: Hana Yamamoto and Hirosaka Inoue.

## CONFLICT OF INTEREST STATEMENT

The authors declare that we have no conflict of interest.

## FUNDING INFORMATION

This research did not receive any specific grants from funding agencies in the public, commercial, or not‐for‐profit sectors.

## ETHICS STATEMENT

The authors have confirmed ethical approval statement is not needed for this submission. Informed consent was obtained from this patient and his families according to our facility's formal forms.

## Data Availability

The dataset generated during the current study are not publicly available but are available from the corresponding author on reasonable request.

